# Expression of *MTAP* Inhibits Tumor-Related Phenotypes in HT1080 Cells via a Mechanism Unrelated to Its Enzymatic Function

**DOI:** 10.1534/g3.114.014555

**Published:** 2014-11-11

**Authors:** Baiqing Tang, Yuwaraj Kadariya, Yibai Chen, Michael Slifker, Warren D. Kruger

**Affiliations:** * Cancer Biology Program, Fox Chase Cancer Center, Philadelphia, Pennsylvania 19111; † Biostatistics Program, Fox Chase Cancer Center, Philadelphia, Pennsylvania 19111

**Keywords:** cell mobility and migration, suppressor genes, methionine

## Abstract

Methylthioadenosine Phosphorylase (*MTAP*) is a tumor suppressor gene that is frequently deleted in human cancers and encodes an enzyme responsible for the catabolism of the polyamine byproduct 5′deoxy-5′-methylthioadenosine (MTA). To elucidate the mechanism by which *MTAP* inhibits tumor formation, we have reintroduced *MTAP* into *MTAP*-deleted HT1080 fibrosarcoma cells. Expression of *MTAP* resulted in a variety of phenotypes, including decreased colony formation in soft-agar, decreased migration, decreased *in vitro* invasion, increased matrix metalloproteinase production, and reduced ability to form tumors in severe combined immunodeficiency mice. Microarray analysis showed that *MTAP* affected the expression of genes involved in a variety of processes, including cell adhesion, extracellular matrix interaction, and cell signaling. Treatment of *MTAP*-expressing cells with a potent inhibitor of MTAP’s enzymatic activity (MT-DADMe-ImmA) did not result in a *MTAP*− phenotype. This finding suggests that *MTAP*’s tumor suppressor function is not the same as its known enzymatic function. To confirm this, we introduced a catalytically inactive version of *MTAP*, D220A, into HT1080 cells and found that this mutant was fully capable of reversing the soft agar colony formation, migration, and matrix metalloproteinase phenotypes. Our results show that *MTAP* affects cellular phenotypes in HT1080 cells in a manner that is independent of its known enzymatic activity.

Methylthioadenosine phosphorylase (MTAP) is a widely expressed metabolic enzyme in the methionine salvage pathway that converts the polyamine byproduct 5′-dideoxy-5′-methylthioadenosine (MTA) into adenine and methylthioribose-1-phosphate (Kamatani and Carson 1981; Olopade *et al.* 1995). Loss of either MTAP protein or the *MTAP* gene is frequent in a large number of different human tumors, including leukemias, lymphomas, mesothelioma, lung carcinoma, pancreatic carcinoma, squamous cell carcinoma, biliary tract cancer, glioblastoma, osteosarcoma, and neuroendocrine tumors (Stadler and Olopade 1996; Dreyling *et al.* 1998; Hori *et al.* 1998; Schmid *et al.* 1998; Wong *et al.* 1998; Brat *et al.* 1999; M’soka *et al.* 2000; Garcia-Castellano *et al.* 2002; Illei *et al.* 2003; Chen *et al.* 2004; Subhi *et al.* 2004; Hustinx *et al.* 2005; Karikari *et al.* 2005). The most frequent mechanism for *MTAP* inactivation is homozygous deletion of the 9p21 region, where both *MTAP* and the *CDKN2A/ARF* tumor suppressor gene complex are located (Nobori *et al.* 1996). Because these deletions generally inactivate *CDKN2A/ARF* as well as *MTAP*, it was initially hypothesized that loss of *MTAP* in tumors was simply due to it being a coincident bystander. However, there is now substantial evidence that *MTAP* itself has tumor suppressor activity. Re-expression of *MTAP* in *MTAP* deleted MCF-7 breast cells results in loss of anchorage-independent growth *in vitro* and loss of tumor formation *in vivo* (Christopher *et al.* 2002). In addition, re-expression of *MTAP* in either a *MTAP*-deleted melanoma cell line or a gastric carcinoma cell line causes reduced cellular invasion *in vitro* (Behrmann *et al.* 2003; Kim *et al.* 2011). Mice heterozygous for a germline deletion of *MTAP* die prematurely of T-cell lymphoma and have accelerated B-cell lymphoma onset when crossed to Eμ-myc mice (Kadariya *et al.* 2009,^2013^). Finally, germline mutations in humans that disrupt primate specific *MTAP* exons are associated with diaphyseal medullary stenosis with malignant fibrous histiocytoma, a rare genetic disease associated with bone dysplasia and cancer (Camacho-Vanegas *et al.* 2012).

The mechanism by which *MTAP* affects tumorigenesis is not understood. Previously, it was shown that *MTAP* expression caused decreased ornithine decarboxylase (ODC) levels and reduced polyamine levels in both yeast and mammalian cells (Subhi *et al.* 2003; Chattopadhyay *et al.* 2005; Tang *et al.* 2006). Because elevated polyamines and ODC are common in cancer cells, it has been speculated that this might be important for *MTAP*’s tumor suppressor function (Subhi *et al.* 2004). A second possible mechanism relates to MTAP’s substrate, MTA. Data from yeast and mammalian cells indicate that loss of *MTAP* results in large elevations of MTA (Kamatani and Carson 1980; Chattopadhyay *et al.* 2006; Stevens *et al.* 2009). Because MTA is a competitive inhibitor of methyltransferase enzymes, including histone and DNA methyltransferases (Williams-Ashman *et al.* 1982), it is possible that loss of *MTAP* may have effects on the epigenetic control of gene expression in tumor cells.

Although the loss of *MTAP* is associated with tumorigenesis, pharmacologic inhibition of MTAP can have antitumor activity. Singh *et al.* have developed a transition-state inhibitor of MTAP, MT-DADMe-ImmA, that binds with extremely high affinity (86 nM K_d_) and completely abolishes enzyme activity (Singh *et al.* 2004). Using this inhibitor, Basu *et al.* (2007) demonstrated that the growth of a *MTAP*+ human head and neck cancer cell line could be inhibited in a xenograft mouse model. Although superficially this seems to contradict the idea that loss of *MTAP* promotes tumorigenesis, it is important to remember that the drug may be exerting its antitumor effects not on the tumor directly but indirectly via its effects on stromal cells. In addition, this antitumor effect was only shown to occur in *MTAP*+ cells not *MTAP*- cells.

In the experiments described here, we have characterized the phenotype of an *MTAP*-deleted HT1080 human fibrosarcoma cell line in which we have stably reintroduced the *MTAP* gene. Our results show that *MTAP* expression inhibits several tumor-related phenotypes and causes global changes in gene expression, affecting several cellular pathways controlling cell adhesion and signaling. However, treatment of these *MTAP*-expressing cells with the MTAP inhibitor MT-DADMe-ImmA, or expression of a mutated version of *MTAP*, did not reverse these effects. Our findings suggest that *MTAP* suppresses tumorigenicity in HT1080 cells via a function that is unrelated to its known enzymatic activity.

## Materials and Methods

### *MTAP*-expressing cell lines

MTAP− (M−), MTAP+ (M+), and D220A cells were created by stably transfecting either the pTRE2:*MTAP*, pTRE2:*MTAP*:D220A, or pTRE2 empty plasmid into HT1080 cells (containing a homozygous *MTAP* deletion) and pooling 10 individual expressing clones together as was previously described (Tang *et al.* 2012). HT1080 cells (Clontech Laboratories, Mountain View, CA) were cultured in Dulbecco’s modified Eagle medium (DMEM) medium supplemented with 2 mM glutamine, 100 μg/mL penicillin, 100 μg/mL streptomycin, 10% fetal bovine serum, and 250 μg/mL G418. Clones were selected using 250 μg/mL hygromycin from a 50 mg/mL stock solution in phosphate-buffered saline (PBS; Sigma-Aldrich, St. Louis, MO). MT-DADMe-ImmA was used at a concentration of 10 μM for all experiments and was obtained from Dr. Vern Schramm (Albert Einstein Medical Center, Bronx, NY). MTA, putrescine, and 2-difluoromethyl-ornithine (DFMO) were obtained from Sigma Aldrich. All media, serum, and antibiotics were obtained from the tissue culture facility at Fox Chase Cancer Center.

### MTAP and ODC activity assay

Protein extracts were prepared from cells lysed in M-PER mammalian protein extraction reagent (Pierce, Rockford, IL) with 1× Complete Mini proteinase inhibitor (Roche Biochemical, Indianapolis, IN) or tissue homogenized by using a dounce homogenizer in PBS containing 10% glycerol with the aforementioned proteinase inhibitor. The extracts were centrifuged at 10,000 × *g* for 15 min at 4°, and the supernatants were collected. Protein concentration was measured with BCA kit (Pierce Rockford, IL). MTAP activity was determined with a photometric assay to measure adenine production as described previously (Christopher *et al.* 2002). ODC activity was assayed by measuring the ^14^CO_2_ formed by decarboxylation of ^14^C-labeled L-ornithine in 30 min at 37° as described previously (Subhi *et al.* 2003). ^14^C-labeled L-ornithine with specificity 5 mCi/mmol was purchased from Moravek Biochemicals (Brea, CA). One unit of MTAP catalyzes the formation of 1 μmol of adenine/mg/minute, whereas 1 unit of ODC catalyzes the formation of 1 nmol of CO_2_/mg/hr.

### Soft agar growth and cell invasion assay

Cells were assessed for growth in soft agar as previously described (Christopher *et al.* 2002). Cells were tested for invasive ability using BD BioCoat Matrigel Invasion chambers (Becton Dickinson, Bedford, MA). Five hundred microliters of media containing 1 × 10^4^ cells was added to each well and incubated for 24 hr at 37° in 5% CO_2_. Cells were fixed and stained with methanol and stained Giemsa. Noninvading cells on the upper surface of the filter were removed by wiping out with a cotton swab, and the filter was excised and mounted on a microscope slide. Invasiveness was quantified by counting cells on the lower surface of the filter.

### Wound healing assay

Cells were inoculated at 2 × 10^5^ per well in 6-well plate and grown to near confluency. They were then scratched with a sterile10-μL pipette tip, rinsed briefly with medium to remove unadhered cells, and reincubated in medium. Wound closure was inspected and photographed at 0, 8, and 24 hr with Nikon Eclipse TS100 microscope and Nikon DXM1200 Digital Camera (Melville, NY).

### Xenograft studies

The M+ and M− cells were transfected with pZsGreen1-N1 vector (Clontech) that expressed ZsGreen1 protein. The green fluorescent ZsGreen1 protein excited at 493 nm and emitted at 505 nm. Because our *MTAP*+ and *MTAP*− cells already expressed neomycin resistance (the marker of the ZsGreen1 vector), a linear puromycin marker (Clontech) was used and cotransfected in a ratio 20:1 (vector: linear marker). Cells were cultured in DMEM medium supplemented with 2 mM glutamine, 100 μg/mL penicillin, 100 μg/mL streptomycin, 10% fetal bovine serum, 250 μg/mL G418, and 1 μg/mL puromycin. Nine individual, puromycin-resistant, high-fluorescent M+ and M− clones were pooled to make the M+ +GFP (M+G) and M− +GFP (M-G) cell lines.

Severe combined immunodeficiency (SCID) mice were subcutaneously injected with 6 × 10^6^ M+G or M−G cells in 200 μL of DMEM. Tumor cell growth was monitored by fluorescent imaging with an IVIS Spectrum Imager (PerkinElmer, Waltham, MA). After 6 wk, mice were killed and tumors were dissected. After the tumor mass was recorded, the tumor was homogenized in PBS with 10% glycerol and 1 x Complete Mini proteinase inhibitor (Roche, Indianapolis, IN) using a dounce homogenizer and MTAP activity was measured.

### Polyamine measurements

Intracellular polyamines were measured on a Biochrom 30 amino acid analyzer using a sodium citrate buffer and a polyamine ion exchange column as previously described (Christopher *et al.* 2002).

### DNA microarray analysis

M+ and M− cells were grown in 100 mM plates until 80% confluent, harvested, and total RNA was extracted using RNeasy Mini Kit (QIAGEN, Valencia, CA) according to manufacturer’s instruction. The quantity and quality of the RNA was assessed with the Agilent 2100 Bioanalyzer. Ten micrograms of total RNA was used to create cDNA by using the One-Cycle cDNA Synthesis kit (Affymetrix, Santa Clara, CA). Biotin-labeled cRNA synthesis was performed using the Affymetrix *In Vitro* Transcription Labeling Kit. The biotinylated cRNA samples were cleaned up, fragmented, and then hybridized to human GeneChip (Human Genome U133 plus 2.0; Affymetrix) in an Affymetrix GeneChip Hybridization Oven 640 according to manufacturer’s protocols. The washing procedures were carried out automatically in Affymetrix GeneChip Fluidics Station 450. The processed GeneChip was scanned with Affymetrix GeneChip Scanner 3000 7G. Highly expressed genes were those determined to have a signal of 50 or greater.

### Statistical analysis

Affymetrix microarray data were analyzed using methods implemented in the R/Bioconductor platform. Raw expression data in the form of Affymetrix CEL files were preprocessed using the Robust Multi-chip Average (RMA) method (Irizarry *et al.* 2003). RMA combines three pre-processing steps: background correction, between-array quantile normalization, and summarization of 25-mer probe intensities into probe set intensity measures.

Differentially expressed genes between groups were identified using the Bioconductor package *limma* (Smyth 2004), which computes empirical Bayes moderated t-statistics for each probe set. This provides more stable comparisons for experiments with small numbers of samples by using information from all probe sets to smooth standard errors. Before differential expression was assessed, a nonspecific filter was applied to reduce the number of probe sets in each comparison. This filter removed all probe sets with low expression across at least 85% of the arrays and those lacking a valid Entrez identifier in the most recent Bioconductor chip annotations. Pairwise comparisons between groups were performed using empirical Bayes moderated two-sample *t*-tests. The Benjamini-Hochberg method was used to adjust *P*-values in order to account for multiple testing (Benjamini and Hochberg 1995). Gene lists for data mining consisted of all probe sets with an adjusted p-value less than 0.01 and at least twofold change between groups. Functional enrichment analysis was performed using the WebGestalt 2.0 software (Wang *et al.* 2013).

### Quantitative real-time polymerase chain reaction (RT-PCR)

Gene-specific probes and primer sets for quantitative Taqman Assays were obtained from Applied Biosystems (Foster, CA). Human β-actin was used as an endogenous normalization for the expression of genes of interest. Quantitative RT-PCR was carried out according to the TaqMan Assay-on-Demand one-step protocol of Applied Biosystems under universal thermal condition in triplicate with ABI-Prism 7900 HT Real Time PCR system.

### Zymography

Extracellular matrix-degrading matrix metalloproteinases (MMPs) present in the cells were subjected to electrophoresis on precast 10% gelatin-containing polyacrylamide gels (Invitrogen, Carlsbad, CA), and their activities detected as a transparent band against a blue background. In brief, cell lysates (25 μg/lane) were mixed with 2X Tris-Glycine SDS sample buffer from Invitrogen (no heating and no addition of reduce reagent), loaded on gels and electrophoresed in 1X Tris-Glycine SDS Running Buffer (Invitrogen) at 125 V and room temperature for about 90 min. After electrophoresis, gels were washed twice with PBS containing 2.5% Triton X-100 for 5 min/each, incubated in 1X Zymogram Renaturing Buffer (Invitrogen) for 45 min, and equilibrated in 1X Zymogram Developing Buffer (Invitrogen) for 35 min at ambient temperature with gentle agitation. The equilibrated gels were incubated in refreshed Zymogram Developing Buffer at 37° overnight or longer for maximum sensitivity and optimal results and stained with SimplyBlue SafeStain (Invitrogen) according manufacturer’s instruction.

### Western blotting

Western blotting was performed as previously described (Christopher *et al.* 2002). Polyclonal rabbit MTAP antibody (Cell Signaling Technology, Danvers, MA) was used at 1:1000 dilution. Monoclonal mouse α-Tubulin antibody (Santa Cruz Biotechnology, Santa Cruz, CA) was used at 1:2000 dilution. After washing, the membranes were incubated with secondary antibody conjugated to horseradish peroxidase at 1:30000 dilution. Membranes were developed using enhanced chemoluminescent reagent (Pierce, Rockford, IL).

### MTA quantitation

Extracts were prepared by sulfocylic acid extraction as previously described (Gupta *et al.* 2009). To remove excess salt, 100 μL of extract was subjected to high-performance liquid chromatography (HPLC) purification using an Agilent 1100 HPLC system (Agilent, Wilmington, DE) containing a Xterra MS C_18_ column (Waters, Milford, MA) and eluted with 25% MeOH and 75% H_2_O.

For LC/MS/MS analysis, a Waters 2690 Alliance HPLC instrument and an LCQ Classic ion trap mass spectrometer (Thermo, San Jose, CA) were used. The purified sample (10 μL) was injected into a Vydak TP C_18_ column (GRACE, Deerfield, IL). The analysis was carried out in isocratic mode, 30% MeOH, 70% H_2_O, and 0.1% formic acid with a flow rate 50 μL/min. The experimental conditions for LCQ were as follows: the spray voltage was set at 5 KV, the sheath gas flow rate was set at 60 (arb), the capillary voltage was set in a range of 20−40 V. Quantitation of MTA was performed by comparing peak area to an internal [5′—^2^H_3_] MTA standard that was synthesized by the in-house organic synthesis facility. The molecular weight of [5′—^2^H_3_] MTA is three units greater than standard MTA. The quantitative measurement of [5′—^2^H_3_] MTA and MTA from the cell lysates and cell media samples was performed by setting the LCQ ion trap mass spectrometer in the selected reaction monitoring scan mode to achieve maximum sensitivity. For MTA, transitions m/z 298.00 → 136.10 and m/z 298.00 → 162.80 were monitored. For [5′—^2^H_3_], transitions m/z 301.10 → 136.10 and m/z 301.10 → 165.90 were monitored. All measurements, including samples for making a calibration curve, triplicate samples from cell lysate, and triplicate samples from cell media, were performed in the same day to avoid potential variance.

## Results

### *MTAP* acts as a tumor suppressor for HT1080 cells

**Figure 1:**
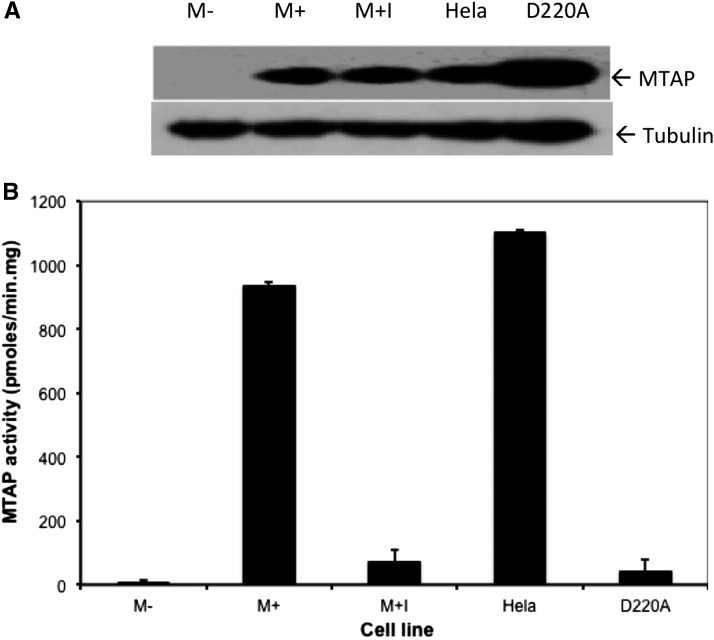
*MTAP* expression and activity in HT1080 cells. (A) Western blot showing levels of MTAP in extracts from stably transfected HT1080 cells. M− is the parent cell line transfected with vector alone (pTRE2). M+ has been transfected with the *MTAP* expressing construct pTRE2:*MTAP*. M+I is the identical to M+, except the cells have been treated for 72 hr with 10 μM the *MTAP* inhibitor, MT-DAD-Me-ImmA. Hela contains extract from a *MTAP*+ Hela cell. D220A contains extract from a HT1080 cell that has been transfected with a plasmid that expresses D220A *MTAP* (pTRE2:*MTAP*:D220A). (B) MTAP enzymatic activity measured in the same extracts as used in (A). Error bars show SD of enzyme assay (n = 4). All means are different from each other as assessed by one-way ANOVA followed by Tukey test (*P* < 0.01 for all comparisons).

HT1080 cells are an immortalized human fibrosarcoma cell line that lacks *MTAP* expression (Tang *et al.* 2000). We created *MTAP*+ (M+) and *MTAP*− (M−) HT1080 cell lines by stably transfecting either a *MTAP* expression plasmid or an empty vector control and pooled multiple clones together to minimize the effects of integration events (Tang *et al.* 2012). The amount of MTAP protein and activity in M+ cells were slightly reduced from those observed in Hela cells (Figure 1, A and B.; compare lane M+ with Hela), indicating that MTAP is being expressed in these cells at near physiologic levels. (We will discuss lanes labeled M+I and D220A in the last two sections.)

**Figure 2:**
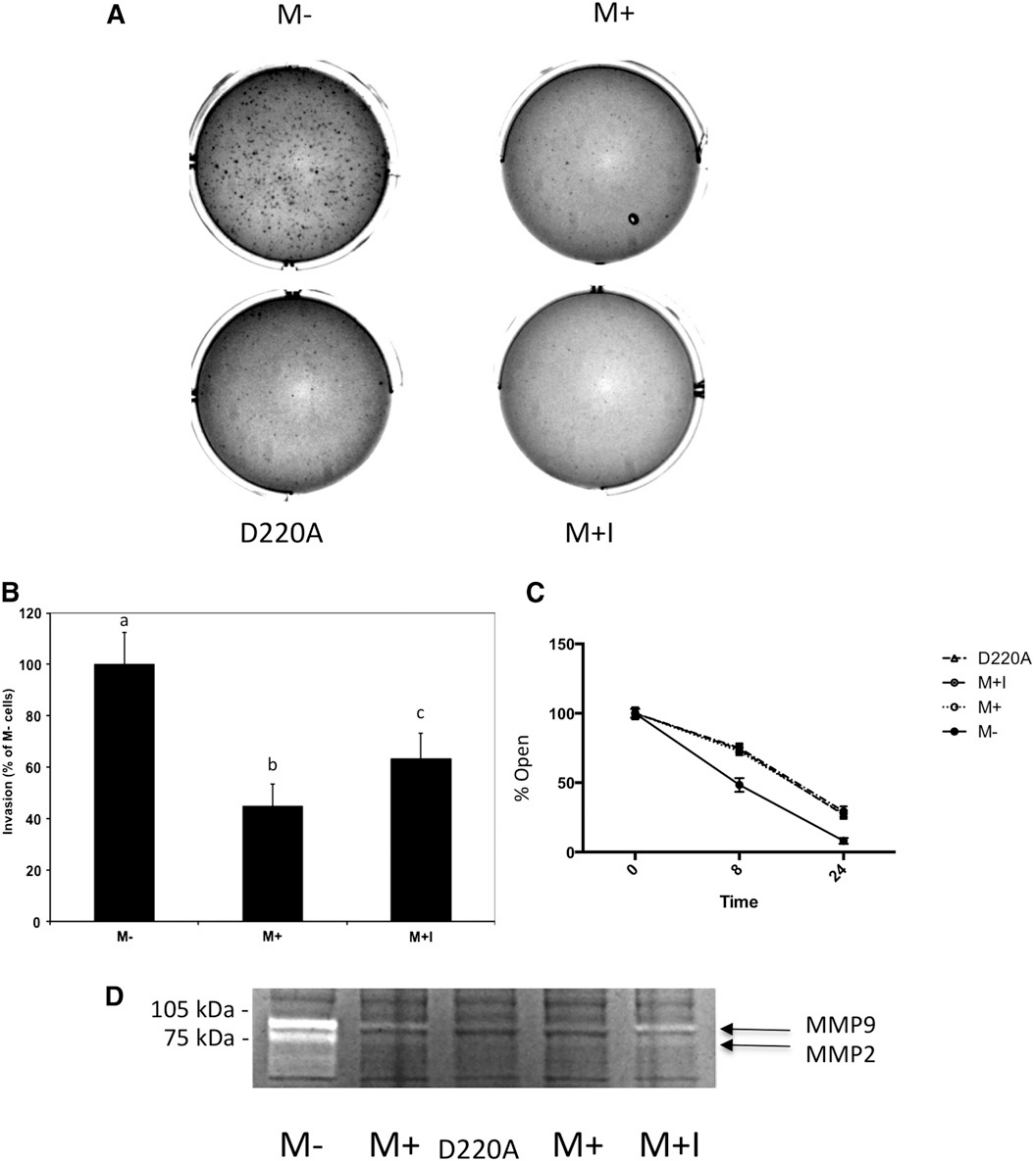
Functional effects of *MTAP* expression in HT1080 cells. (A) Growth of indicated cell lines in soft agar. Ten thousand cells were plated in each well and image was taken after 14 d. Labeling is identical to Figure 1. (B) Invasion of cells through matrigel. Indicated cells are expressed as a percentage of the M− line. All assays performed in nine separate wells (n = 9). SD is indicated by bars. Different letters above each bar indicates that the means of all columns are different from each other as assessed by one-way ANOVA followed by Tukey test (*P* < 0.01 for all comparisons). (C) MTAP expression and wound healing. Confluent monolayers of the indicated cells were scratched and wound healing was monitored at 0, 8, and 24 hr as described in the *Materials and Methods*. Results are expressed as percent of wound openness. Error bars show SEM (n = 6). (D) Cell extracts (50 μg) from the indicated strains were loaded onto 10% gelatin-containing polyacrylamide gels and stained as described in the *Materials and Methods*. White bands indicate where MMPs have digested gelatin.

We performed several assays to examine the effects of MTAP expression on tumor-related phenotypes. Under standard tissue culture conditions, we observed no difference in doubling time between M+ and M− cells (25.6 ± 0.8 *vs.* 24.8 ± 0.8 hr, *P* = ns) and no obvious differences in overall cellular morphology (Supporting Information, Figure S1). However, we did observe a dramatic decrease in the ability of M+ cells to form colonies on soft agar (Figure 2A). In addition, we found that the M*+* cells exhibited a 55% reduction (*P* < 1 × 10^−6^) in migration through a basement membrane preparation (Matrigel) using a Boyden chamber assay compared with M− cells (Figure 2B). We also found that M− cells showed increased mobility in a wound-healing assay (Figure 2C).

Because MMPs have been shown to be important for invasion through matrigel, we examined metalloprotease activity. This was done using gel zymography, a technique in which gelatin is embedded in the gel and metalloprotease activity is detected by digestion of the gelatin. These studies found that *MTAP* expression greatly inhibited both MMP-9 and MMP-2 activity (Figure 2D). Quantitative RT-PCR of MMP-9 message indicates that this down-regulation is occurring at the level of mRNA (Figure S2). These findings show that expression of *MTAP* can inhibit cellular functions related to migration and invasion.

**Figure 3:**
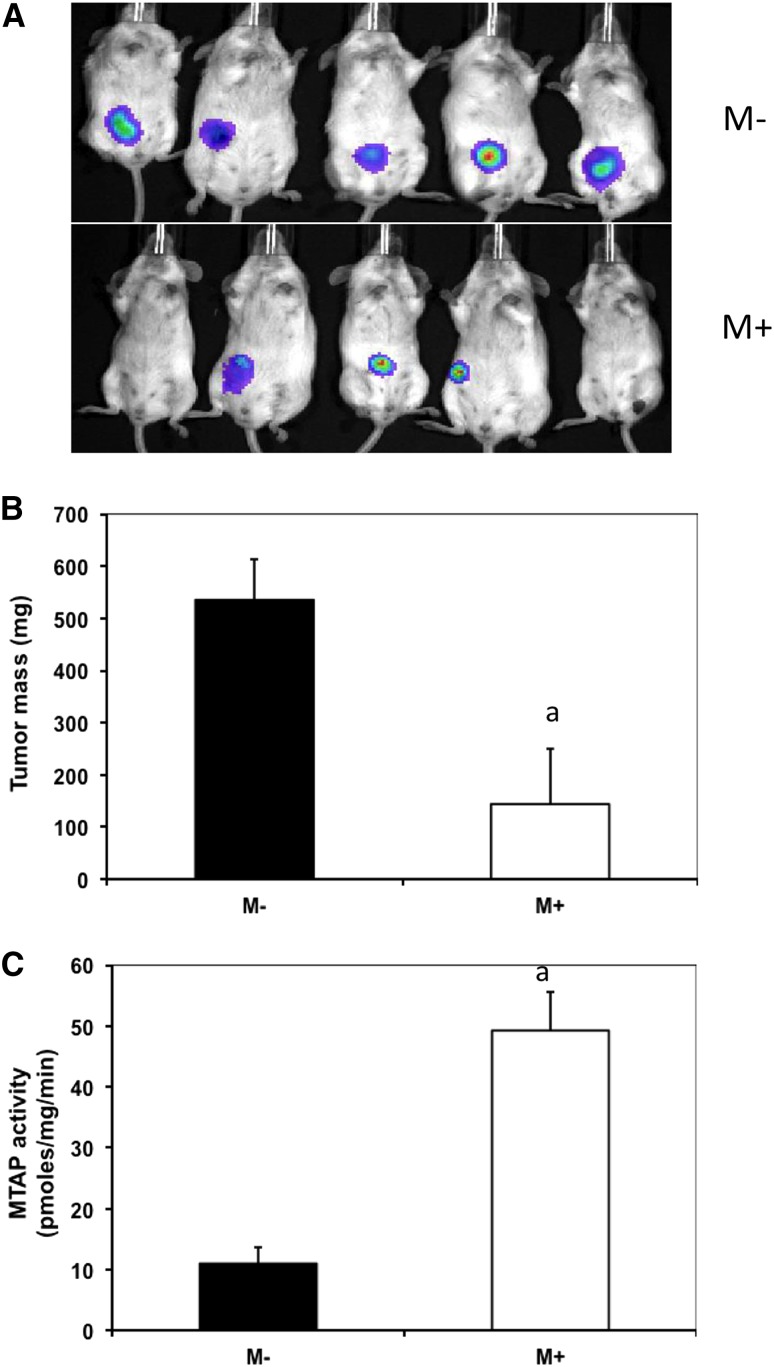
Growth of M− and M+ cells in SCID mice. (A) Green-florescent labeled HT1080 cells (6 × 10^6^) of the indicated genotype were injected into the flank of SCID mice (n = 5/group) and imaged after 6 wk using an in vivo imaging system imager. (B) Weight of tumors at 6 wk. Error bars show SD. Letter “a” indicates significant difference (*P* < 0.02) between cell types (C) *MTAP* enzymatic activity of tumors. Letter “a” indicates significant difference (*P* < 0.003) between cell types.

Finally, we injected M+ and M− cells expressing a ZsGreen1 protein tag (see the section *Materials and Methods*) into SCID mice to determine whether *MTAP* expression could affect tumor cell growth in a mouse xenograft model. Four of the five mice injected with M+ cells had reduced tumor burden after 4 wk compared with mice injected with M− cells as judged by the level of ZsGreen1 protein fluorescence (Figure 3A). In addition, the mean weight of the excised tumor was reduced by 73% (Figure 3B). To confirm the MTAP status of the cells, we also measured MTAP activity in the excised tumors (Figure 3C). We found that the tumors excised from mice injected with M− cells had a 70% reduction in MTAP activity, indicating that the majority of cells in the tumor were derived from the injected M− cells (Figure 3C). These findings show that *MTAP* expression inhibits the tumor formation of HT1080 cells in mice.

### *MTAP* expression alters HT1080 gene expression profile

To identify the molecular pathways responsible for MTAP’s antitumorigenic phenotypes, we performed RNA microarray profiling using Affymetrix arrays. Using a criteria of at least a twofold difference in expression and a false discovery rate <0.01, we identified 283 probe sets that were up-regulated and 64 probe sets that were down-regulated by *MTAP*, between M+ and M− cells (Table S1). As there were 17,475 probe sets expressed above background on the array, this means that 2% of the probes were at least twofold differentially regulated. This list of 347 probes identified a total of 254 unique transcripts, which were then examined to see whether they were enriched in any particular functional pathways. Using the KEGG pathway database, we found enrichment for genes in several pathways involved in cell adhesion, cell communication, and cell migration (Table S2). The pathways identified are consistent with our functional observations that loss of *MTAP* affects colony formation in soft-agar, cell migration, and expression of MMPs. Interestingly, several genes specific to the Wnt-signaling pathway were identified. This pathway has been shown to control process involved in cell migration, adhesion, and differentiation (Neth *et al.* 2007; Guo *et al.* 2008).

**Figure 4:**
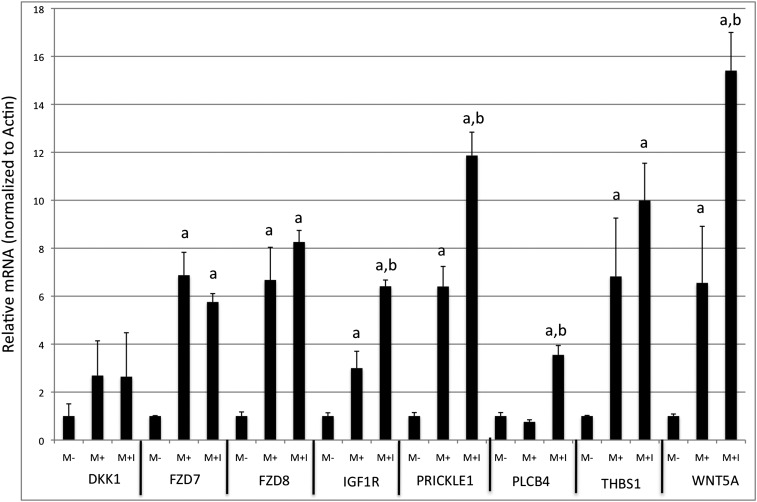
Quantitative real-time polymerase chain reaction (RT-PCR) of selected genes. The indicated genes were quantified by RT-PCR in M−, M+, or M+ cells exposed to MT-DAD-Me-ImmA (M+I) for 48 hr (see the section *Materials and Methods*). Data show relative transcript level compared with M− cells. All reactions were done in triplicate, and the SE is indicated. Letter “a” indicates a significant difference (*P* < 0.05) between each column and M− cells, whereas “b” indicates significant difference between M+ and M+I cells.

To confirm the microarray results, quantitative RT-PCR was used to examine the RNA levels of eight of the genes identified as having altered regulation by *MTAP*. These genes were selected because they were identified as being involved in Wnt or other signaling pathways (Figure 4). Six of the selected genes were found to be up-regulated, and two were down-regulated on the microarray. We found that seven of the eight genes tested confirmed the findings of the microarray analysis.

### *MTAP* expression does not affect polyamine or ODC activity in HT1080 cells

Previously, our group reported that expression of *MTAP* in MCF-7 breast adenocarcinoma cells also repressed soft agar growth and tumor formation in mice. In these cells, *MTAP* expression resulted in decreased ODC activity and decreased levels of intracellular polyamines (Christopher *et al.* 2002). Because ODC is a known oncogene (Gerner and Meyskens 2004), these findings suggested a possible mechanism for *MTAP*’s tumor suppressor effects. To see whether this also occurred in HT1080 cells, we measured intracellular polyamine levels and ODC activity in the M*+* and M*−* cells (Figure S3). Unlike MCF-7 cells, we did not observe any significant differences in either polyamine or ODC activity. In addition, although soft-agar colony formation in MCF-7 cells was inhibited by DFMO (an irreversible inhibitor of ODC activity) and stimulated by putrescine (the product of the ODC reaction), we failed to see any such effects in HT1080 cells (Figure S4). These results show that in HT1080 cells, *MTAP* expression does not exert its phenotypic effects by modulation of ODC or polyamine levels.

### MTA accumulation does not explain *MTAP*’s phenotypic effects

**Figure 5:**
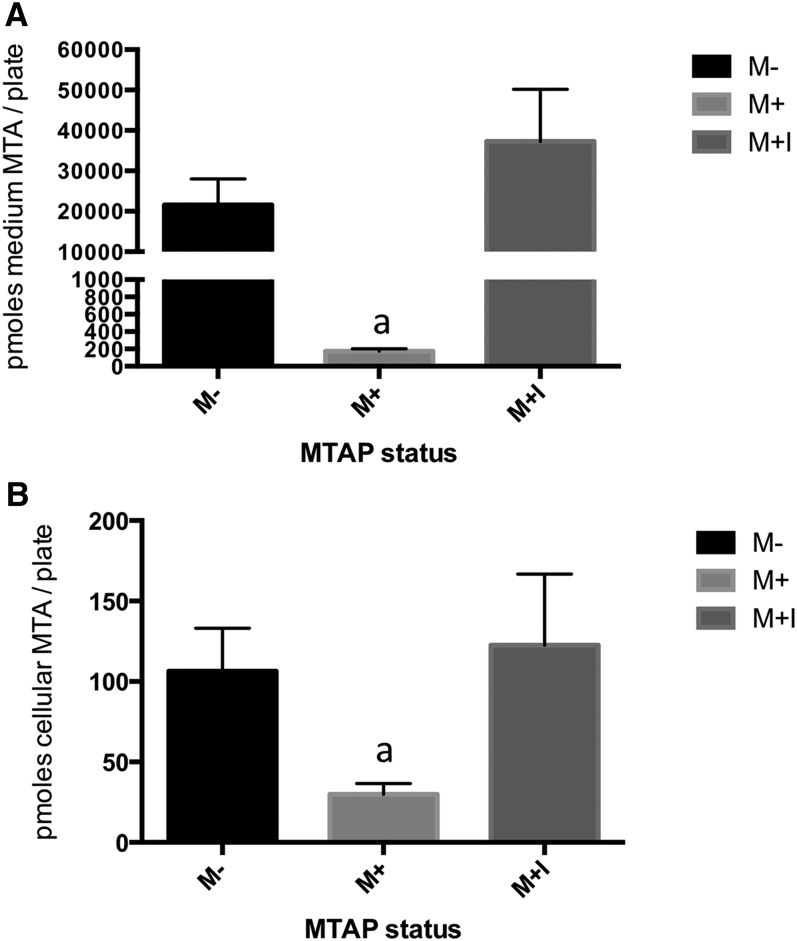
MTA levels in M−, M+, and M+I cells. (A) MTA levels as measured in cell medium from the indicated cell lines. Mean and SD are shown for each of the indicated cell lines (n = 3). “a” indicates *P* < 0.005 compared with M− cells. (B) Same as above, but MTA is measured in cells.

An alternative hypothesis to explain the effects of *MTAP* expression on cellular phenotypes involves the accumulation *MTAP*’s substrate, MTA. MTA is known to inhibit a variety of methyltransferase enzymes including histone and DNA methyltransferases (Williams-Ashman *et al.* 1982), and exogenous MTA added to either melanoma or hepatocellular carcinoma cell lines can alter expression of *MMP* and growth factor genes (Stevens *et al.* 2009; Kirovski *et al.* 2011). Therefore, it seemed possible that this might be the mechanism by which *MTAP* could influence the RNA expression levels of a large number of genes. To test this idea, we treated M+ cells with MT-DAD-Me-ImmA, a drug that is a transition state analog and is a potent inhibitor of MTAP enzymatic activity (Singh *et al.* 2004). To confirm that the compound was actually inhibiting MTAP function, we measured enzyme activity in MT-DAD-Me-ImmA−treated cells (M+I, Figure 1B) and found that drug treatment resulted in a 93% reduction in enzyme activity. We also examined both intra- and extracellular MTA levels using LC-MS/MS in M−, M+, and M+I cells. As expected, M+ cells had very low levels of both intracellular and extracellular MTA compared with M− cells, showing that the presence of MTAP dramatically affects MTA levels (Figure 5A). In M+I cells, we found that both intracellular and extracellular MTA levels were actually slightly greater than those observed in M− cells. These findings indicate that the MT-DAD-Me-ImmA is effectively inhibiting MTAP activity and causing significant MTA accumulation.

**Figure 6:**
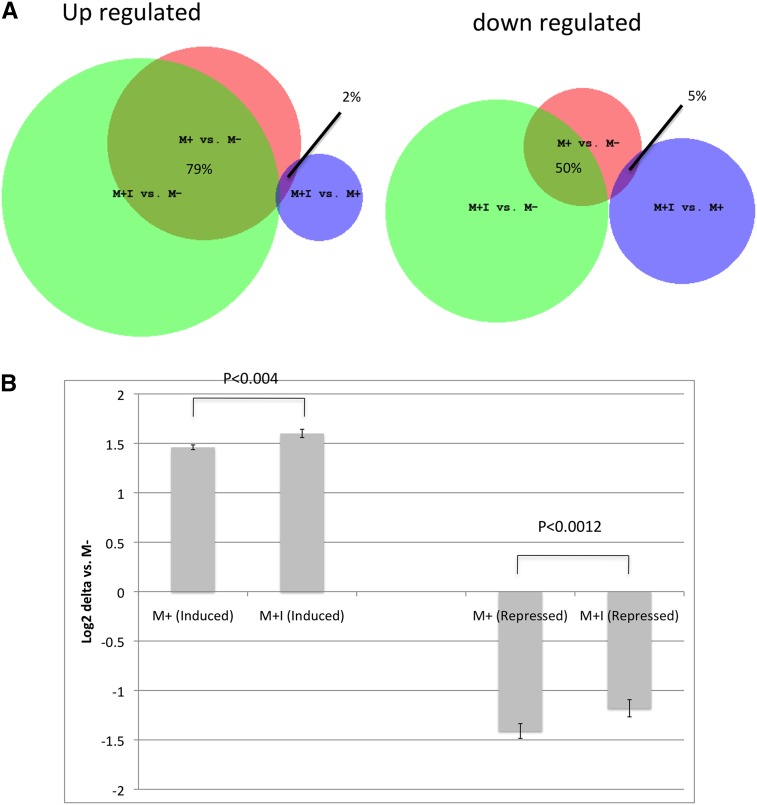
Comparison of transcriptional profiles between M−, M+, and M+I cells. (A) Venn diagram showing overlap in differentially regulated genes from the indicated comparisons. Size of circle is proportional to the number of differentially regulated genes in each comparison. The percentage of overlap between the different comparisons involving the M+ *vs.* M− set is shown. (B) Comparison of mean induction or repression of differentially expressed genes in M+, and M+I cells *vs.* M− controls. The error bars show the SEM. *P*-values are also shown (paired *t*-test, two-sided).

We next compared the gene expression profiles in all three cell lines (M−, M+, and M+I). Our expectation was that M+ cells treated with the inhibitor would show gene expression profiles similar to M− cells. Thus, we expected that a comparison of M+I *vs.* M+ differentially expressed genes would have substantial overlap with the M− *vs.* M+ list. However, this was not the case. Only one (2%) of the M− *vs.* M+ up regulated genes were found in the M+I *vs.* M+ up-regulated gene set, and only three (5%) of the down-regulated genes were in common (Figure 6A). In contrast, the gene expression pattern in M+I cells was much more similar to M+ cells, with 79% of the up-regulated genes and 50% of the down-regulated genes being in common. To explore this further, we compared the mean fold-difference in gene expression in all 347 probe sets that were identified as either up or down regulated in M+ cells relative to M− to the mean fold difference observed in the M− *vs.* M+I comparison (Figure 6B). For induced genes, the mean log_2_ fold change was 1.45 in M− *vs.* M+ compared with 1.52 in M- *vs.* M+I. For repressed genes, the mean log_2_ fold change was 1.37 in M− *vs.* M+ compared with 1.03 in M− *vs.* M+I. These results show that the addition of the inhibitor did not greatly affect the ability of *MTAP* to affect the mRNA levels of downstream genes. We also observed the similar effects on the quantitative PCR of the selected differentially regulated signaling genes shown in Figure 4. These results indicate that majority of the downstream transcriptional effects of MTAP occur even when its enzymatic activity is inhibited.

To control for any nonspecific effects of the inhibitor, we also performed a separate array experiment in which we compared M− cells and M− cells plus inhibitor. We did not observe any genes that were differentially expressed using the same selection criteria (twofold difference, false discovery rate < 0.01). These results show that the inhibitor is quite specific for MTAP, and that the differences observed between M+ and M+I cells are due to MTAP inhibition.

Finally, we examined the effect of MT-DAD-Me-ImmA on several different functional assays related to tumor formation. MT-DAD-Me-ImmA treatment of M+ cells failed to promote growth in soft-agar (Figure 2A) and did not reverse the inhibitory effect of MTAP on wound healing (Figure 2C). However, MT-DAD-Me-ImmA treatment did cause a slight increase in MMP-9 and MMP2 levels (Figure 2D) and some increase in invasion potential (Figure 2B).

### Mutant *MTAP* expression has similar effects as wild type *MTAP* in HT1080 cells

Aspartate 220 is a critical residue in the *MTAP* catalytic site that is necessary for enzymatic activity. Previously, our laboratory had shown that D220A *MTAP* failed to suppress soft-agar colony formation and elevated polyamine levels in *MTAP*-deleted MCF-7 cells (Christopher *et al.* 2002). Therefore, we decided to test the affects of this mutation in HT1080 cells. As described in the Methods section, we created a novel HT1080 cell line, D220A that stably expresses D220A *MTAP*. Western analysis shows that the mutant protein is abundantly expressed, but has less than 3% of the enzyme activity present in cells expressing wild-type *MTAP* (Figure 1). These cells were then examined using three functional assays: growth in soft agar, wound healing, and MMP-2 and MMP-9 activity (Figure 2, A and C). In all of these assays, D220A cells behaved identically to M+ cells. These findings support the view that *MTAP*’s tumor suppressor function in HT1080 cells does not require its known enzyme activity.

## Discussion

In the experiments described here, we have assessed the consequences of ectopic *MTAP* expression on an *MTAP* deleted human fibrosarcoma−derived cell line. *MTAP* expression suppressed the ability of these cells to grow in SCID mice, their ability to grow on soft agar, and their ability to invade through a collagen matrix. All of these phenotypes support the idea that *MTAP* is a tumor suppressor gene and are consistent with our previous study that *MTAP* acted as a tumor suppressor gene in a MCF-7 breast cancer cell line (Christopher *et al.* 2002).

At the molecular level, we found that *MTAP* expression in HT1080 cells resulted in the significant alteration of the gene expression profile. Specifically, *MTAP* affected the expression of genes involved in a number of important cellular functions including cell adhesion, cell communication, and cell migration. Our findings are consistent with reports that *MTAP* expression in gastric carcinoma cells inhibits migration in a wound-healing assay, and that inhibition of *MTAP* causes increased expression of MMP-1 and MMP-9 in a human hepatocellular carcinoma cell line (Kim *et al.* 2011). Given the effects of *MTAP* expression on invasion, migration, and soft agar growth, it was particularly interesting that *MTAP* affected the expression of several genes involved in the Wnt-signaling pathway. The Wnt-signaling pathway frequently is found activated in a variety of human cancers and is thought to play a key role in a variety of developmental processes related to cell migration and epithelial to mesenchyme transition (Neth *et al.* 2007). In our studies, several of the Wnt-signaling transcripts were elevated when MTAP was expressed. However, it is unclear whether this reflects increased Wnt-signaling or a response to lower Wnt-signaling. Further studies will be needed to clarify this point.

The key finding of this study was that *MTAP* protein, but not enzymatic activity, was required for *MTAP*’s tumor suppressor effects on HT1080 cells. Inhibition of *MTAP*’s enzymatic activity with a potent transition state inhibitor failed to significantly reverse any of the functional effects of *MTAP* expression, despite effectively inhibiting MTAP’s enzyme activity in cell extracts and causing the accumulation of MTA. This finding is not consistent with the idea that MTA inhibition of histone and DNA methyltransferases are behind *MTAP*’s effect on tumorigenesis. In addition, expression of a catalytically inactive mutant form of the enzyme (D220A) was able to suppress soft-agar colony formation and MMP-2 and MMP-9 expression. This particular result was unexpected given our previous finding that this same mutant was not effective in suppressing tumor formation in MCF-7 cells (Christopher *et al.* 2002), and the findings of Kirovski and Stevens that exogenous MTA can up-regulate growth factors and MMPs in melanoma and hepatocellular carcinoma cell lines (Kirovski *et al.* 2011; Stevens *et al.* 2009). However, there are some important differences between these experiments and the ones reported here. In MCF-7 cells, *MTAP* expression reduces the steady-state levels of polyamines and causes a significant decrease in ODC activity, whereas in HT1080 cells, neither of these effects is observed. Furthermore, in MCF-7 cells soft-agar colony formation is inhibited by DFMO and stimulated by putrescine, but this also was not observed in HT1080 cells. With regards to the data reported by Bosserhoff *et al.*, it is important to note that although there is clearly an effect of exogenous MTA on gene expression and various functional assays, the effects are relatively modest, and it is unclear whether these affects alone are sufficient to explain all of the biological effects of *MTAP*-loss. Taken in total, these findings suggest that *MTAP* may exert its tumor suppressor effects via two different mechanisms; an enzyme-dependent mechanism that may involve the accumulation of the *MTAP* substrate MTA, and a nonenzymatic mechanism that predominates in HT1080 cells.

What are the possible nonenzymatic functions of *MTAP*? Global proteomic studies carried out in *MTAP*^+^ Hela cells have identified 20 proteins that appear to form complexes with *MTAP* (Table S3). These include proteins that are involved in a variety of different molecular functions including vesicle trafficking, purine metabolism, transcription/chromatin regulation, cytoskeletal function, and RNA metabolism. Interestingly, the ortholog of *MTAP* in *Saccharomyces cerevisiae*, *MEU1*, was initially identified in a mutant screen specifically designed to identify genes involved in transcriptional regulation (Donoviel and Young 1996). These findings are all consistent with the idea that *MTAP* may have a nonenzymatic function that may involve interactions with other proteins. Future studies will need to focus on the elucidation of these functions.

In summary, the studies described here show that *MTAP* acts as a tumor suppressor gene in HT1080 cells, affecting functions related to cell adhesion, cell communication, and cell invasion. *MTAP* expression causes a large change in the cells gene expression profile, but this effect is not dependent on *MTAP*’s known enzymatic function. Our results show that *MTAP* has additional nonenzymatic functions that play an important role in its tumor suppressor function.

## Supplementary Material

Supporting Information
