# Supplementary material for: Expression of MTAP Inhibits Tumor-Related Phenotypes in HT1080 Cells via a Mechanism Unrelated to Its Enzymatic Function
Source: G3 (Bethesda). 2014 Nov 11;5(1):35–44. doi: 10.1534/g3.114.014555 (PMC4291467; doi:10.1534/g3.114.014555)
Supplement: Supporting Information [file supp_5_1_35__index.html]

Expression of MTAP Inhibits Tumor-Related Phenotypes in HT1080 Cells via a Mechanism Unrelated to Its Enzymatic Function — Supporting Information 

# Expression of *MTAP* Inhibits Tumor-Related Phenotypes in HT1080 Cells via a Mechanism Unrelated to Its Enzymatic Function

## Supporting Information for Tang *et al.*, 2015

**Files in this Data Supplement:**

- Supporting Information - Figures S1-S4 and Tables S1-S3 (PDF, 1 MB)
- Figure S1 - Morphology of different cell lines used in this study. (PDF, 761 KB)
- Figure S2 - MMP9 mRNA levels in M+ and M- cells treated with exogenous methylthioadenosine. (PDF, 535 KB)
- Figure S3 - Polyamine and ODC activity of MTAP+ and MTAP- cells. (PDF, 464 KB)
- Figure S4 - Affect of DFMO and putrescine on HT1080 cells. (PDF, 605 KB)
- Table S2 - Pathways enriched in M+ vs. M-. (PDF, 7 KB)
- Table S3 - Putative MTAP interacting proteins. (PDF, 134 KB)
- Table S1 - .xlsx, 50 KB
