# Supplementary material for: Expression of MTAP Inhibits Tumor-Related Phenotypes in HT1080 Cells via a Mechanism Unrelated to Its Enzymatic Function
Source: G3 (Bethesda). 2014 Nov 11;5(1):35–44. doi: 10.1534/g3.114.014555 (PMC4291467; doi:10.1534/g3.114.014555)
Supplement: Supporting Information [file supp_g3.114.014555_014555SI.pdf]

G3·Genes | Genomes | Genetics

Expression of *MTAP* Inhibits Tumor Related Phenotypes in HT1080 Cells via a Mechanism Unrelated to its Enzymatic Function

Baiqing Tang[*], Yuwaraj Kadariya[*], Yibai Chen[†], Michael Slifker[†], and Warren D. Kruger[*, 1]

Authors' Affiliations: [*]Cancer Biology Program, [†]Biostatistics Program, Fox Chase Cancer Center, Philadelphia, Pennsylvania 19111

[1]**Correspondence:** Warren D. Kruger, Fox Chase Cancer Center, 333 Cottman Avenue Philadelphia, PA 19111 Phone: 215-728-3030 Fax: 215-214-1623

Email: warren.kruger@fccc.edu

Microarray data has been deposited at Gene Expression Omnibus (GSE56112).

DOI: 10.1534/g3.114.014555

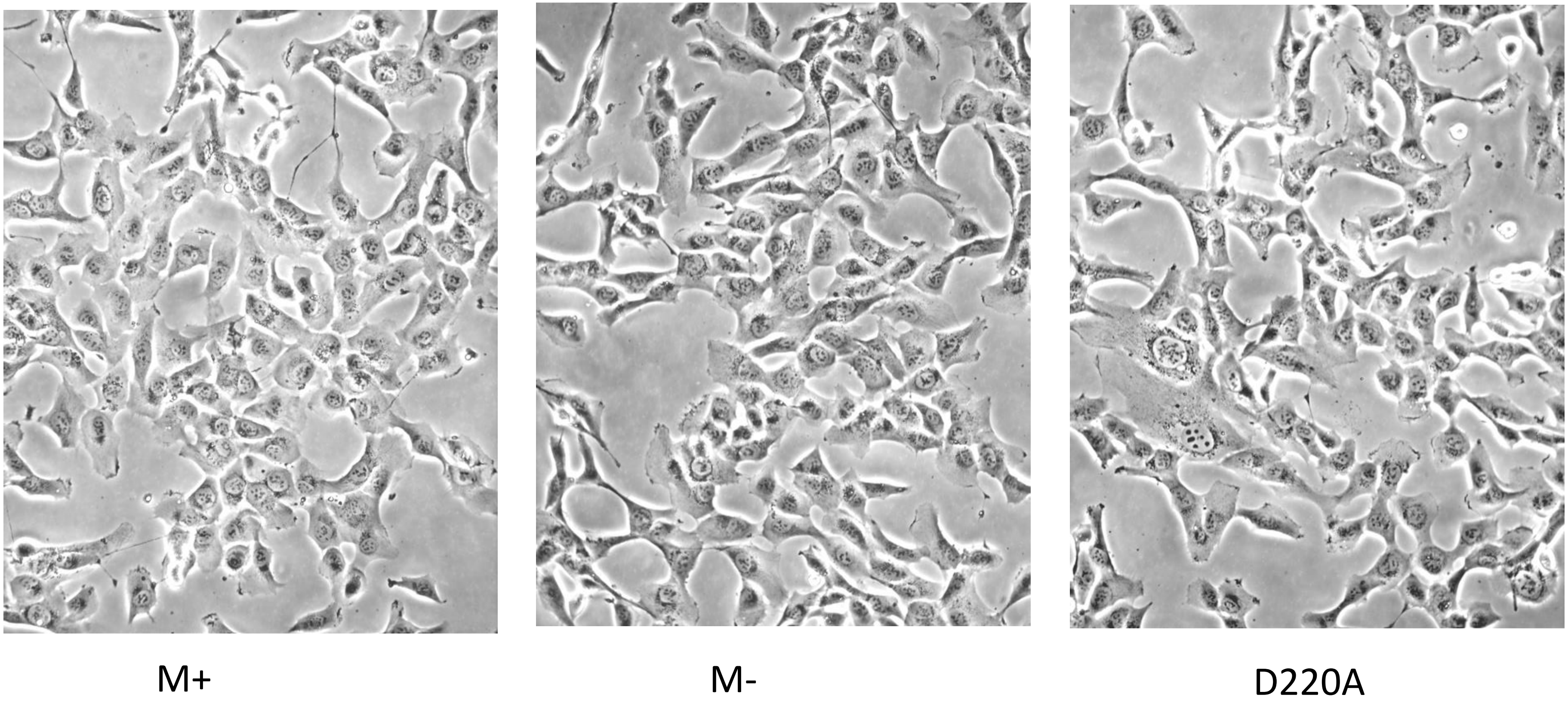

Figure S1 Morphology of different cell lines used in this study. Photos are at 200x magnification.

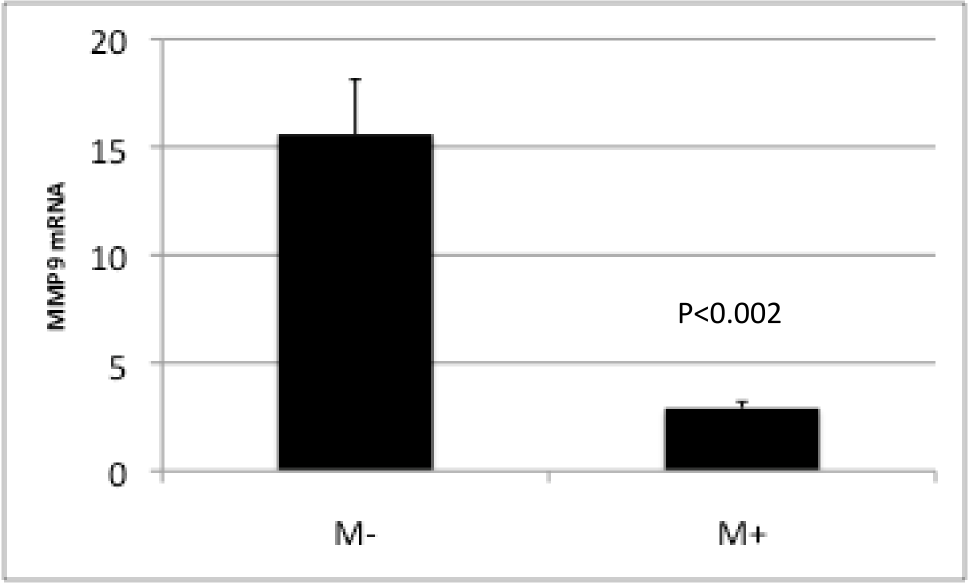

Figure S2 MMP9 mRNA levels in M+ and M- cells treated with exogenous methylthioadenosine. $2x10^7$ M- and M+ cells were seeded in 10 cm dishes and grown for 24h at which time MTA was added as indicated. The cells then incubated for an additional 48 hours and then RNA was isolated. MMP9 mRNA was quantified using Taqman probes as described in methods. All samples were assessed in triplicate and standard deviation is shown.

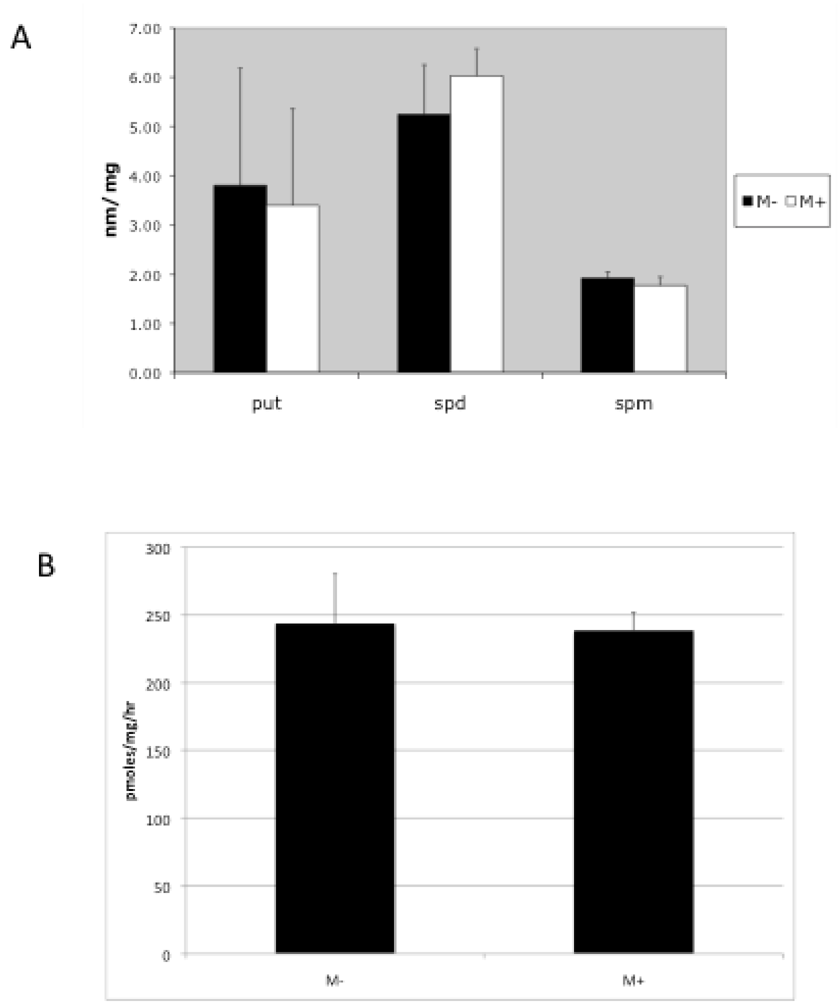

Figure S3 Polyamine and ODC activity of MTAP+ and MTAP- cells. A. Intracelluar concentration of putrescine, spermidine, and spermine. Error bars show standard deviation (n=3). B. ODC activity. Error bars show standard deviation (n=3).

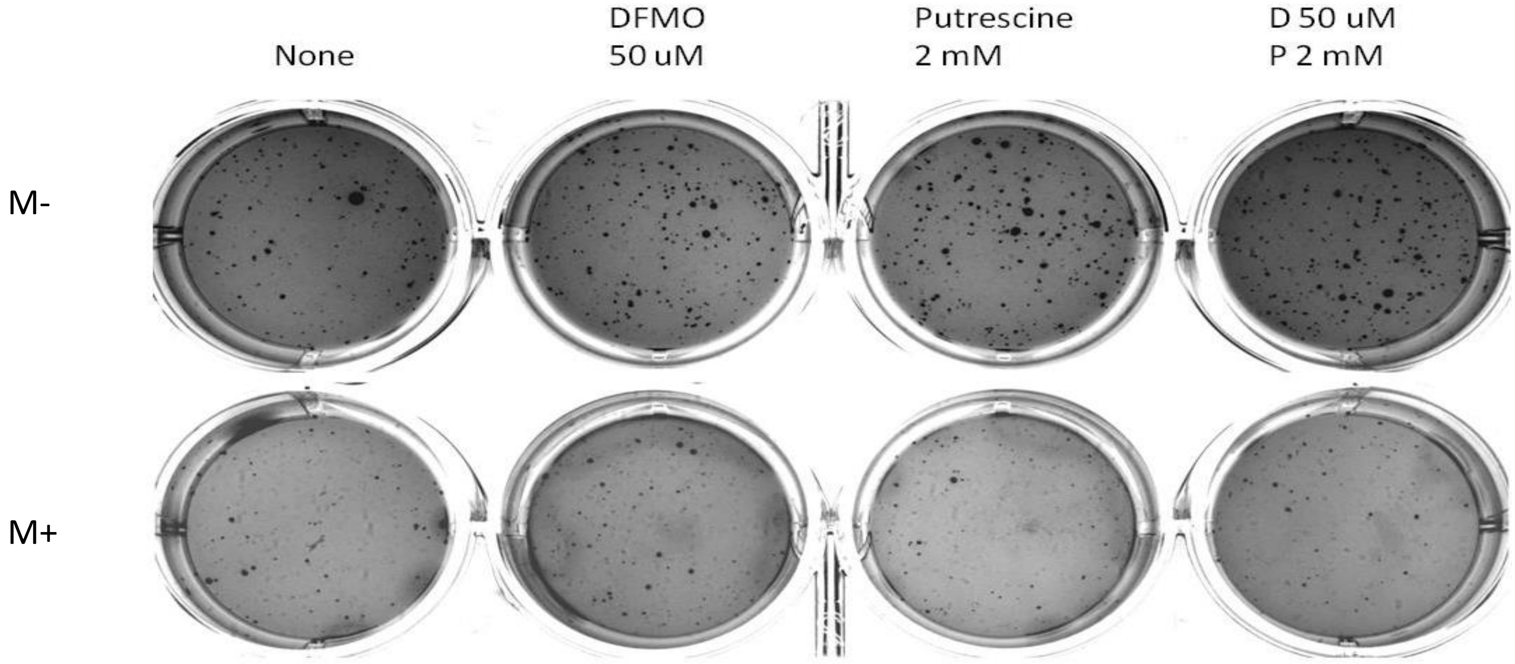

Figure S4 Affect of DFMO and putrescine on HT1080 cells. Indicated cells were grown in soft agar as described in methods. Either nothing, DFMO (D), Putrescine (P), or both were added as indicated. Pictures were taken after 16 days.

Table S1 is available for download as an Excel file at http://www.g3journal.org/lookup/suppl/doi:10.1534/g3.114.014555/-/DC1

Table S2 Pathways enriched in M+ vs. M-

KEGG pathway	Genes up down regulated	Enrichment
ECM-receptor interaction	FN1; ITGA10; THBS2; ITGB5; COL6A1; THBS1; SDC2	C=41; O=7; E=1.02; R=6.91, rawP=$5.67x10^{-5}$; adjP=0.0006
Cytokine-cytokine receptor interaction	IL15; TNFRSF21; KITLG; VEGFA; TGFBR2; TNFRSF19	C=64;O=6;E=1.58;R=3.8;rawP=0.0048; adjP=0.0264
Focal Adhesion	IGF1R; FN1; THBS2; COLA6; ITGA10; VEGFA; ITGB5; THBS1	C=126; O=8; E=3.11; R=2.57; rawP=0.0128; adjP=0.0286
Malanogenesis	PLCB4; KITLG; FZD7; FZD8; WNT5A	C=57; O=5; E=1.41; R=3.55; rawP=0.0130; adjP=0.0286
Wnt-signaling pathway	PRICKLE1; FZD7; TBL1X; FZD8; DAAM1; DKK1;WNT5A	C=95; O=7; E=2.35; R=2.98; rawP=0.009; adjP=0.0286

C: Total number of genes, O: observed gene number in particular pathway; E: expected number of genes in a specific pathway for an interesting gene set; R: ration of enrichment (O/E); P: significance of enrichment calculated from hypergeometric test.

Table S3 Putative MTAP interacting proteins.

Gene	Protein	Function	Technique
UBC	Ubiquitin	ubiquitin	AC
ELAVL1	ELAV-like Protein	RNA binding	AC
ANXA6	Annexin VI	ER transport	AC
ATP6V1A	Vacuolar ATPase	ER transprot	AC
CUTA	cutA divalent cation tolerance homologue	Membrane	AC
CFL1	Cofilin	Cytoskelatal	AC
GOT1	Glutamic-oxaloacetic transaminase	AA metabolism	AC
UBE2H	Ubiquitin E2	ubiquitin	AC
CHRAC1	chromatin accessiblity complex 1	transcription	AC
GMPS	GMP synthase	purine metab	AC
HPRT1	Hypoxanthine-guanine phosphoribosyltransferase	purine metab	AC
PNP	Purine nucleotide phosphorylase	purine metab	AC
UROD	uroporphyrinogen decarboxylase	heme metab	AC
MTPN	Myotrophin	transcription	AC
PDIA3	Protein disulfide isomerase family A	ER transprot	AC
RAB1A	Ras-like G-protein	ER transprot	AC
CDC25A	CDC25 phosphatase	Cell cycle	2H
FOLH1	Glutamate carboxypeptidase 2	purine metab	2H
RELB	v-rel reticuloendotheliosis viral oncogene homolog B	transcription	2H
LYRM1	LYR motif containing 1	unknown	2H

All interactions are taken from (http://thebiogrid.org/110611/table/homo-sapiens/mtap.html). AC indicates that the interaction was identified in a high throughput affinity Capture-MS experiment, while 2H indicates that it was observed in a high throughput 2-hybrid screen.
