# Supplementary material for: Expression of MTAP Inhibits Tumor-Related Phenotypes in HT1080 Cells via a Mechanism Unrelated to Its Enzymatic Function
Source: G3 (Bethesda). 2014 Nov 11;5(1):35–44. doi: 10.1534/g3.114.014555 (PMC4291467; doi:10.1534/g3.114.014555)
Supplement: Supporting Information [file supp_g3.114.014555_FigureS1.pdf]

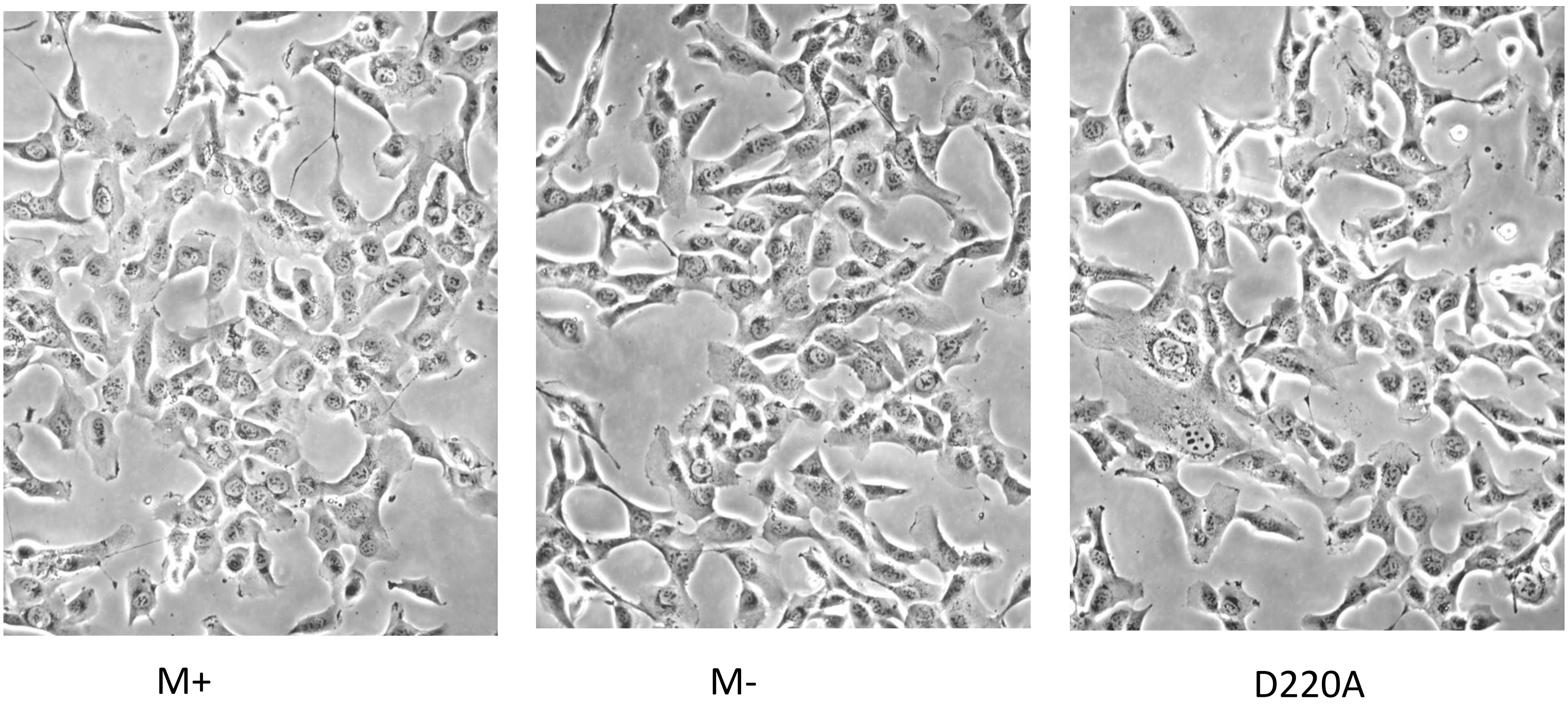

Figure S1 Morphology of different cell lines used in this study. Photos are at 200x magnification.
