# Supplementary material for: Expression of MTAP Inhibits Tumor-Related Phenotypes in HT1080 Cells via a Mechanism Unrelated to Its Enzymatic Function
Source: G3 (Bethesda). 2014 Nov 11;5(1):35–44. doi: 10.1534/g3.114.014555 (PMC4291467; doi:10.1534/g3.114.014555)
Supplement: Supporting Information [file supp_g3.114.014555_FigureS2.pdf]

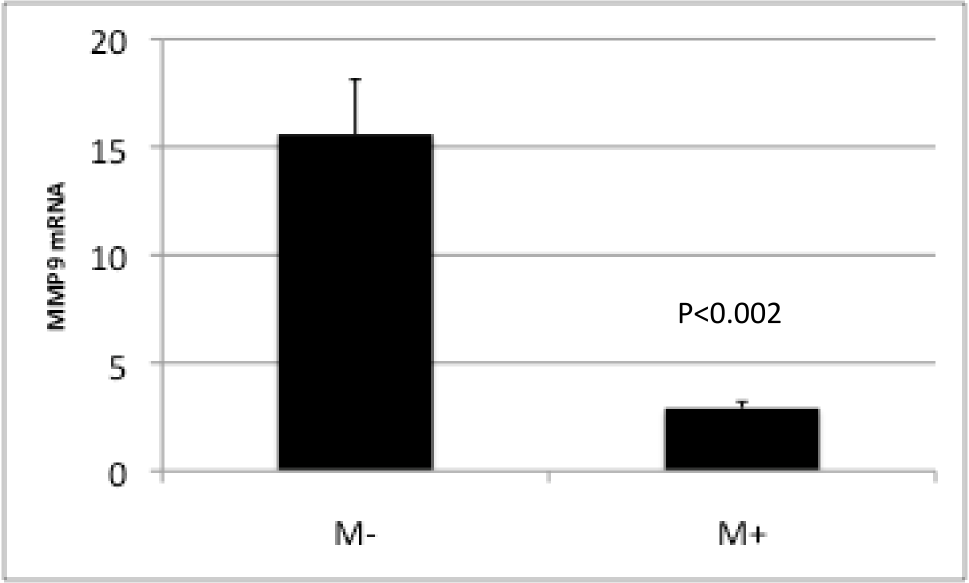

Figure S2 MMP9 mRNA levels in M+ and M- cells treated with exogenous methylthioadenosine. $2x10^7$ M- and M+ cells were seeded in 10 cm dishes and grown for 24h at which time MTA was added as indicated. The cells then incubated for an additional 48 hours and then RNA was isolated. MMP9 mRNA was quantified using Taqman probes as described in methods. All samples were assessed in triplicate and standard deviation is shown.
