# Supplementary material for: Expression of MTAP Inhibits Tumor-Related Phenotypes in HT1080 Cells via a Mechanism Unrelated to Its Enzymatic Function
Source: G3 (Bethesda). 2014 Nov 11;5(1):35–44. doi: 10.1534/g3.114.014555 (PMC4291467; doi:10.1534/g3.114.014555)
Supplement: Supporting Information [file supp_g3.114.014555_FigureS3.pdf]

A

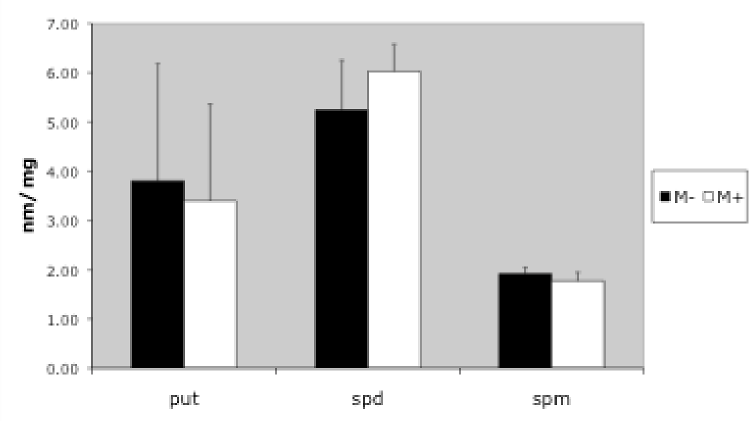

B

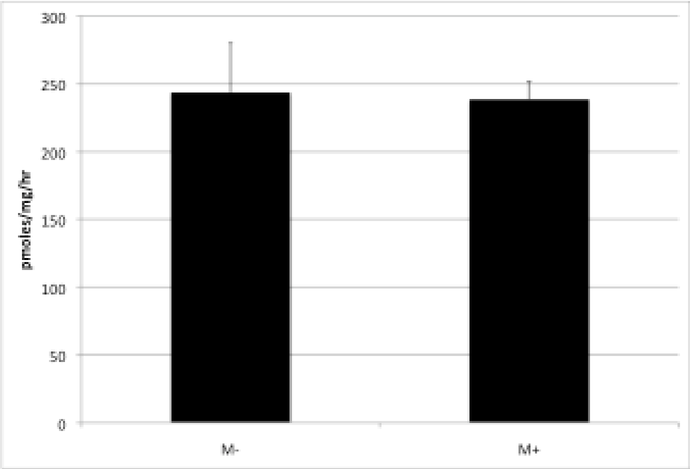

Figure S3 Polyamine and ODC activity of MTAP+ and MTAP- cells. A. Intracelluar concentration of putrescine, spermidine, and spermine. Error bars show standard deviation (n=3). B. ODC activity. Error bars show standard deviation (n=3).
