# Supplementary material for: Expression of MTAP Inhibits Tumor-Related Phenotypes in HT1080 Cells via a Mechanism Unrelated to Its Enzymatic Function
Source: G3 (Bethesda). 2014 Nov 11;5(1):35–44. doi: 10.1534/g3.114.014555 (PMC4291467; doi:10.1534/g3.114.014555)
Supplement: Supporting Information [file supp_g3.114.014555_FigureS4.pdf]

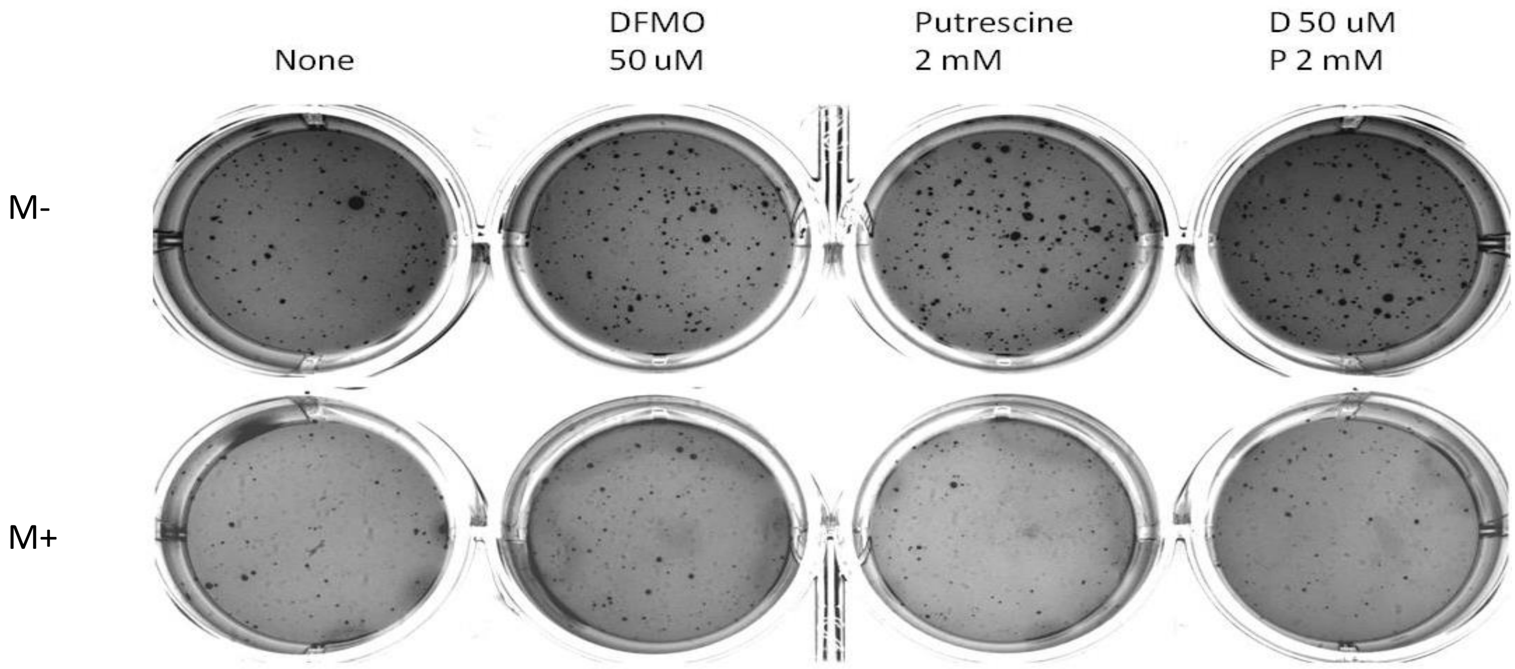

Figure S4 Affect of DFMO and putrescine on HT1080 cells. Indicated cells were grown in soft agar as described in methods. Either nothing, DFMO (D), Putrescine (P), or both were added as indicated. Pictures were taken after 16 days.
