# Supplementary material for: Expression of MTAP Inhibits Tumor-Related Phenotypes in HT1080 Cells via a Mechanism Unrelated to Its Enzymatic Function
Source: G3 (Bethesda). 2014 Nov 11;5(1):35–44. doi: 10.1534/g3.114.014555 (PMC4291467; doi:10.1534/g3.114.014555)
Supplement: Supporting Information [file supp_g3.114.014555_TableS2.pdf]

Table S2 Pathways enriched in M+ vs. M-

KEGG pathway	Genes up down regulated	Enrichment
ECM-receptor interaction	FN1; ITGA10; THBS2; ITGB5; COL6A1; THBS1; SDC2	C=41; O=7; E=1.02; R=6.91, rawP=5.67x10^{-5}; adjP=0.0006
Cytokine-cytokine receptor interaction	IL15; TNFRSF21; KITLG; VEGFA; TGFBR2; TNFRSF19	C=64;O=6;E=1.58;R=3.8;rawP=0.0048; adjP=0.0264
Focal Adhesion	IGF1R; FN1; THBS2; COLA6; ITGA10; VEGFA; ITGB5; THBS1	C=126; O=8; E=3.11; R=2.57; rawP=0.0128; adjP=0.0286
Malanogenesis	PLCB4; KITLG; FZD7; FZD8; WNT5A	C=57; O=5; E=1.41; R=3.55; rawP=0.0130; adjP=0.0286
Wnt-signaling pathway	PRICKLE1; FZD7; TBL1X; FZD8; DAAM1; DKK1;WNT5A	C=95; O=7; E=2.35; R=2.98; rawP=0.009; adjP=0.0286

C: Total number of genes, O: observed gene number in particular pathway; E: expected number of genes in a specific pathway for an interesting gene set; R: ration of enrichment (O/E); P: significance of enrichment calculated from hypergeometric test.
