# Supplementary material for: Expression of MTAP Inhibits Tumor-Related Phenotypes in HT1080 Cells via a Mechanism Unrelated to Its Enzymatic Function
Source: G3 (Bethesda). 2014 Nov 11;5(1):35–44. doi: 10.1534/g3.114.014555 (PMC4291467; doi:10.1534/g3.114.014555)
Supplement: Supporting Information [file supp_g3.114.014555_TableS3.pdf]

Table S3 Putative MTAP interacting proteins.

Gene	Protein	Function	Technique
UBC	Ubiquitin	ubiquitin	AC
ELAVL1	ELAV-like Protein	RNA binding	AC
ANXA6	Annexin VI	ER transport	AC
ATP6V1A	Vacuolar ATPase	ER transprot	AC
CUTA	cutA divalent cation tolerance homologue	Membrane	AC
CFL1	Cofilin	Cytoskelatal	AC
GOT1	Glutamic-oxaloacetic transaminase	AA metabolisn	AC
UBE2H	Ubiquitin E2	ubiquitin	AC
CHRAC1	chromatin accessiblity complex 1	transcription	AC
GMPS	GMP synthase	purine metab	AC
HPRT1	Hypoxanthine-guanine phosphoribosyltransferase	purine metab	AC
PNP	Purine nucleotide phosphorylase	purine metab	AC
UROD	uroporphyrinogen decarboxylase	heme metab	AC
MTPN	Myotrophin	transcription	AC
PDIA3	Protein disulfide isomerase family A	ER transprot	AC
RAB1A	Ras-like G-protein	ER transprot	AC
CDC25A	CDC25 phosphatase	Cell cycle	2H
FOLH1	Glutamate carboxypeptidase 2	purine metab	2H
RELB	v-rel reticuloendotheliosis viral oncogene homolog B	transcription	2H
LYRM1	LYR motif containing 1	unknown	2H

All interactions are taken from (http://thebiogrid.org/110611/table/homo-sapiens/mtap.html). AC indicates that the interaction was identified in a high throughput affinity Capture-MS experiment, while 2H indicates that it was observed in a high throughput 2-hybrid screen.
